# How does the level of care change after proximal humeral fracture in older patients? A propensity score matching analysis

**DOI:** 10.1007/s41999-026-01531-w

**Published:** 2026-06-26

**Authors:** Jeanette Koeppe, Janette Iking, Karen Fischhuber, Jan P. Happe, Ursula Marschall, Michael J. Raschke, Josef Stolberg-Stolberg, J. Christoph Katthagen

**Affiliations:** 1 Institute of Biostatistics and Clinical Research, University of Muenster, Schmeddingstrasse 56, 48149 Muenster, Germany; 2 Department of Epidemiology and Biostatistics, Public and Global Health, Medical University Lausitz – Carl Thiem, Thiemstraße 111, 03048 Cottbus, Germany; 3 Research Group “Mathematical Surgery”, University Hospital Muenster, University of Muenster, Muenster, Germany; 4 Department of Trauma-, Hand- and Reconstructive Surgery, University Hospital Muenster, Albert-Schweitzer-Campus 1, Building W1, 48149 Muenster, Germany; 5 BARMER Institute for Health System Research, Lichtscheider Strasse 89, 42285 Wuppertal, Germany

**Keywords:** Proximal humeral fracture, Level of care, Geriatric surgery, Real world evidence, Reverse total shoulder arthroplasty, Locked-plate fixation

## Abstract

**Purpose:**

In the context of an aging population, the impact of surgical versus non-operative treatment for proximal humeral fractures (PHFs) on level of care (LoC) in older adults remains poorly understood.

**Methods:**

This retrospective study analyzed claims data from the German BARMER health insurance fund, including patients aged ≥ 65 years with PHF (ICD S42.2) between 01/2017 and 09/2022. Patients were categorized into non-operative or surgical treatment groups, with surgery further divided into locked-plate fixation (LPF), reverse total shoulder arthroplasty (RTSA), and other. A 1:1 propensity score matching adjusted for age, sex, pre-fracture LoC, year of fracture, and risk profile was performed. Primary endpoints were overall survival, LoC deterioration, and nursing home care. Hazard ratios were estimated using Cox proportional hazards or Fine and Gray models to account for competing risks.

**Results:**

Among 54,595 patients (median age 79, 84% female), 44.3% underwent surgery. After matching (31,314 patients), 21.3% of patients experienced LoC deterioration one year after PHF. RTSA was associated with increased risk of LoC worsening (HR 1.40; 95% CI 1.31–1.50), while LPF showed no difference compared to non-operative treatment. Risk for progression to severe care dependency (LoC ≥ 3) or nursing home care did not differ between treatment groups. Surgical treatment was associated with reduced overall mortality (HR 0.88; 95% CI 0.84–0.91).

**Conclusion:**

While RTSA was associated with a higher risk of LoC deterioration, differences in severe care dependency were not observed. Surgical treatment was linked to lower mortality, supporting individualized treatment decisions that consider both survival and long-term care outcomes.

**Level of evidence:**

Level III, retrospective comparative study.

**Supplementary Information:**

The online version contains supplementary material available at https://doi.org/10.1007/s41999-026-01531-w.

## Key summary points

**Aim:**

To investigate the association between surgical versus non-operative treatment of proximal humeral fractures and long-term care dependency and survival in older adults using nationwide German health insurance data.

**Findings:**

Reverse total shoulder arthroplasty was associated with a higher risk of deterioration in level of care one year after fracture, whereas locked-plate fixation showed no difference compared with non-operative treatment. Surgical treatment overall was associated with lower all-cause mortality, and no treatment strategy differed with respect to progression to severe care dependency or nursing home admission.

**Message:**

Treatment decisions for proximal humeral fractures in older adults should consider potential effects on long-term care dependency alongside survival, supporting individualized, geriatric-oriented decision-making.

**Supplementary Information:**

The online version contains supplementary material available at https://doi.org/10.1007/s41999-026-01531-w.

## Introduction

Proximal humeral fractures (PHFs) represent the third most common fracture type among older people and are frequently associated with functional decline, frailty, and loss of independence [1, 2]. While the majority of patients are treated non-operatively, locked-plate fixation (LPF) and reverse total shoulder arthroplasty (RTSA) are the most frequently performed surgical interventions [3]. Current evidence does not clearly favor one procedure over another, although RTSA may yield superior outcomes compared to LPF [4–11]. However, most existing studies primarily focus on radiological or shoulder-specific functional outcomes, whereas patient-centered outcomes, such as autonomy, care dependency, and long-term functional trajectories remain insufficiently explored.

Surgical treatment may facilitate early functional mobilization, which represents a key determinant of recovery and prevention of secondary geriatric complications, and recent data indicate lower rates of major adverse and thromboembolic events [3, 4]. Therefore, careful patient selection is crucial, with particular attention to expected functional recovery and independence in daily life. Especially in older adults, treatment decisions must consider not only fracture characteristics but also frailty, multimorbidity, cognitive status, and baseline functional capacity. Given the high prevalence of PHF among older adults and its potential consequences, understanding its impact on long-term care needs is essential – not only from a clinical perspective but also in terms of the patients' quality of life and preservation of autonomy as a central goal of geriatric medicine. Accordingly, the aim of this study was to determine whether specific treatment strategies are associated with a reduced risk of requiring a higher level of care following PHF, or not.

Germany faces substantial socio-economic challenges, with one–third of its population aged 65 years or older as of 2022 [12]. Demographic projections estimate that the number of individuals requiring long-term care will rise to between 5.6 and 14 million by 2050 [13]. As a result, hospitalization rates and treatment durations are expected to increase steadily, potentially causing public health care expenditures of the German federal state to nearly triple [14, 15]. From a geriatric perspective, fractures in older adults represent sentinel events that may accelerate trajectories of dependency and institutionalization, thereby posing major challenges to long-term care systems. To implement effective preventive measures and demand-oriented care structures, it is essential to understand the long-term impact of specific fracture treatments in older patients, particularly regarding subsequent care needs and nursing home dependency.

In Germany’s statutory long-term care insurance (LTCI) system, the need for support in daily living is organized into five levels of care (LoC), ranging from minor impairments of autonomy (LoC I) to the most severe form of dependency (LoC V) [16]. Benefits include financial support, training programs, consultation services, nursing care, and housekeeping assistance. Since the LTCI’s introduction in 1995, the number of benefit recipients has increased fivefold, and further growth is anticipated [17]. While increases in LoC following hip fractures are well documented [18, 19], data on PHFs remain limited, and existing studies often do not differentiate between treatment modalities [20, 21]. To our knowledge, this is the first nationwide German study to systematically assess how different treatment strategies for PHF influence trajectories of long-term care dependency in older adults.

## Methods

### Data pool and patient cohort

As described in prior studies, the remuneration system in Germany is based on the “German Diagnosis-Related Groups” (G-DRG) system, which is specified and regulated by mandatory coding instructions, including encoded diagnoses (International Statistical Classification of Diseases, German Modification; ICD-10 GM) and procedures (German operation and procedure classification; *Operationen- und Prozedurenschlüssel,* OPS) [3, 22–24]. As for anonymity of insurance data, no prior written individual informed consent for the analyzed data had to be obtained.

For the study at hand, patient remuneration data (inpatient and outpatient data) from the BARMER health insurance from 2005 to 2021 were available. All older patients (age ≥ 65 years) with in- or outpatient coded PHF (ICD S42.2) between 01/2017 and 09/2022 were included in the study (*n* = 54,595), with exclusion criteria outlined in Supplementary Figure S1. Patients with a prior PHF within five years were excluded. Treatment allocation was defined within the first 21 days after fracture, and patients with shorter follow-up time were excluded (*N* = 1203). With a median follow-up of 37.5 months, patients were observed from final treatment allocation (i.e., 21 days after PHF) to the end of follow-up (defined as the study end date 31.12.2022, exit from database, or death from any cause). Patients were grouped into non-operative and surgical treatment, with surgical treatment further subdivided into locked-plate fixation for simple and multi-fragmented fractures (sLPF/LPF; OPS 5-793.k1, 5-793.31; 5-794.21, 5-794.k1), reverse total shoulder arthroplasty (RTSA; OPS 5-824.21), and other surgical treatments (see Suppl. Table S1, including non-assignable procedures). Comorbidities and prior pharmaceutical treatment were identified within 24 months prior to PHF. All definitions for all variables and endpoints are presented in Supplementary Table S1.

### Pre-existing LoC and the German LTCI system

Pre-existing LoC was detected within two years before PHF, using the latest reported level. In Germany, the statutory long-term care insurance (LTCI) system defines five levels of care (“Pflegegrade”), ranging from Level I (minor impairment) to Level V (most severe impairment). For more details on the German LTCI system see Additional Information 1 in the Supplemental Material.

### Primary endpoints

Primary endpoints were defined as overall survival (OS), LoC ≥ I, LoC ≥ II, LoC ≥ III, deterioration of care level (with and without death), and the need for nursing home care (NHC).

### Missing values

Except for missing information on basic demographic data, such as sex, date of birth, and date of death (defined as exclusion criteria), no missing data occurred in the study, as all variables were defined by existing ICD or OPS codes. If no related code was found, the variable was set to zero.

### Propensity score matching

**Fig. 1:**
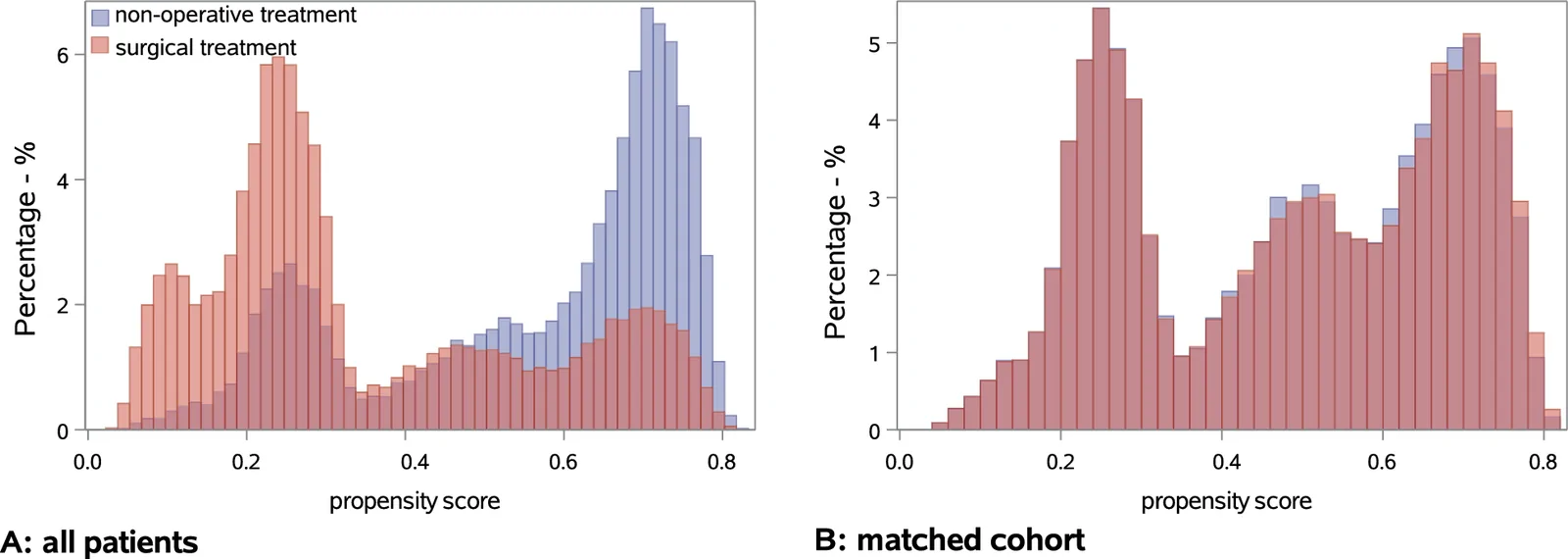
Overlap between treatment groups before (**A**) and after 1:1 propensity score matching (**B**). Propensity score (ps) was determined using multivariable logistic regression to model the probability of surgical treatment including year of fracture and all factors that are presented in Table 1. Overlap is presented by the histogram of logit (ps) depending on treatment group

To adjust for differences between treatment groups, a 1:1 greedy nearest neighbor propensity score matching without replacement was performed, including variables such as age, sex, pre-existing LoC, year of fracture, and patients’ risk profile (see Table 1). Overlap before and after matching is presented in Fig. 1. Hyperparameters for the matching algorithm were chosen in such a way that the sum of squared z differences (SSQZ) was minimized around half the number of variables included in the matching procedure (k), i.e.,

$${\raise0.7ex\hbox{$k$} \!\mathord{\left/ {\vphantom {k 2}}\right.\kern-0pt} \!\lower0.7ex\hbox{$2$}} \approx SSQZ .$$

**Table 1:** Propensity score matching with all co-variables. Patient characteristics, risk differences and Z differences to evaluate balance between matching groups

	Before matching		After matching			
	Control: non-operative treatment	Surgical treatment	Control: non-operative treatment	Surgical treatment	Risk difference (95% CI)	Z difference
Frequency – *n* (%)	30,391 (55.7%)	24,204 (44.3%)	15,657 (50.0%)	15,657 (50.0%)	n.a	n.a
First diagnosis in outpatient sector – *n* (%)	18,663 (61.4%)	5081 (21.0%)	5075 (32.4%)	5074 (32.4%)	-0.0% (-1.0 – 1.0%)	0.324
Nursing home before PHF – n (%)	2886 (9.5%)	1178 (4.9%)	1106 (7.1%)	1113 (7.1%)	0.0% (-0.5 – 0.6%)	0.071
Previous LoC – *n* (%) No LoC	19,656 (64.7%)	18,008 (74.4%)	10,354 (66.1%)	10,354 (66.1%)	n.a	n.a
LoC I	713 (2.4%)	641 (2.7%)	383 (2.5%)	383 (2.5%)		
LoC II	3881 (12.8%)	2457 (10.2%)	2091 (13.4%)	2091 (13.4%)		
LoC III	3528 (11.6%)	2037 (8.4%)	1832 (11.7%)	1832 (11.7%)		
LoC IV	2060 (6.8%)	875 (3.6%)	837 (5.4%)	837 (5.4%)		
LoC V	553 (1.8%)	186 (0.8%)	160 (1.0%)	160 (1.0%)		
Mean hospital frailty risk score (± SD)	13.7 (± 12.0)	12.0 (± 10.7)	13.6 (± 11.5)	13.5 (± 11.7)	-0.06 (-0.32–0.29)	-0.462
Mean age – years (± SD)	79.5 (± 8.2)	78.3 (± 7.5)	79.4 (± 8.1)	79.1 (± 7.7)	-0.25 (-0.42–0.07)	-2.776
Mean index year – years (± SD)	2019.5 (± 1.7)	2019.5 (± 1.6)	2019.5 (± 1.7)	2019.5 (± 1.6)	0.02 (-0.02–0.05)	0.914
Female sex – *n* (%)	25,225 (83.0%)	20,620 (85.2%)	13,100 (83.7%)	13,151 (84.0%)	0.3% (-0.5–1.1%)	0.783
Osteoporosis – *n* (%)	10,475 (34.5%)	8630 (35.7%)	5556 (35.5%)	5556 (35.5%)	0.0% (-1.1–1.1%)	0.000
Prior osteoporosis-assoc. fractures within 5 years before PHF – *n* (%)	6975 (23.0%)	5613 (23.2%)	3869 (24.7%)	3890 (24.8%)	0.1% (-0.8–1.1%)	0.247
Cancer – *n* (%)	8815 (29.0%)	6775 (28.0%)	4510 (28.8%)	4509 (28.8%)	-0.0% (-1.0–1.0%)	0.288
Diabetes mellitus – *n* (%)	9243 (30.4%)	7266 (30.0%)	4814 (30.8%)	4845 (30.9%)	0.2% (-0.8–1.2%)	0.379
Dementia – *n* (%)	4618 (15.2%)	2537 (10.5%)	2178 (13.9%)	2154 (13.8%)	-0.2% (-0.9–0.6%)	-0.393
Chronic polyarthritis – *n* (%)	2028 (6.7%)	1584 (6.5%)	1051 (6.7%)	1046 (6.7%)	-0.0% (-0.6–0.5%)	-0.113
Obesity – *n* (%)	6204 (20.4%)	5,00 (22.3%)	3304 (21.1%)	3326 (21.2%)	0.1% (-0.8–1.1%)	0.304
Nicotine abuses – *n* (%)	2190 (7.2%)	2019 (8.3%)	1195 (7.6%)	1242 (7.9%)	0.3% (-0.3–0.9%)	0.991
Parkinson – *n* (%)	1227 (4.0%)	857 (3.5%)	625 (4.0%)	639 (4.1%)	0.1% (-0.4–0.5%)	0.402
Alcohol abuses – *n* (%)	1364 (4.5%)	1393 (5.8%)	817 (5.2%)	855 (5.5%)	0.2% (-0.3–0.7%)	0.052
Omarthrosis – *n* (%)	1178 (3.9%)	674 (2.8%)	496 (3.2%)	488 (3.1%)	-0.1% (-0.4–0.3%)	-0.259
Frozen shoulder – *n* (%)	1307 (4.3%)	863 (3.6%)	597 (3.8%)	593 (3.8%)	-0.0% (−0.5–0.4%)	-0.118
Atrial fibrillation/flutter – *n* (%)	6374 (21.0%)	4,550 (18.8%)	3353 (21.4%)	3,330 (21.3%)	-0.2% (-1.1–0.8%)	-0.317
Congestive heart failure – *n* (%)	7501 (24.7%)	5400 (22.3%)	3961 (25.3%)	3936 (25.2%)	-0.2% (-1.1–0.8%)	-0.312
Coronary heart disease – *n* (%)	7781 (25.6%)	5499 (22.7%)	3916 (25.0%)	3895 (24.9%)	-0.1% (-1.1–0.8%)	-0.275
Hypertension – *n* (%)	25,015 (82.3%)	20,068 (82.9%)	13,052 (83.4%)	13,084 (83.6%)	0.2% (-0.6–1.0%)	0.487
Atherosclerosis – *n* (%)	5450 (17.9%)	4222 (17.4%)	2827 (18.1%)	2841 (18.2%)	0.1% (-0.8–0.9%)	0.205
Stroke and/or CVD before PHF – *n* (%)	8825 (29.0%)	6312 (26.1%)	4452 (28.4%)	4428 (28.3%)	-0.2% (-1.2–0.9%)	-0.301
Chronic kidney disease – *n* (%)	8319 (27.4%)	6415 (26.5%)	4490 (28.7%)	4488 (28.7%)	-0.0% (-1.0–1.0%)	-0.025
Any anticoagulant – *n* (%)	7489 (24.6%)	5241 (21.7%)	5386 (34.4%)	5363 (34.3%)	-0.2% (-1.2–0.9%)	-0.274
Any anti-osteoporotic medication – *n* (%)	3978 (13.1%)	3123 (12.9%)	2026 (12.9%)	2,056 (13.1%)	0.2% (-0.6–0.9%)	0.504
Compl. within 21 days after diagnosis – *n* (%) Major adverse events	671 (2.2%)	597 (2.5%)	464 (3.0%)	443 (2.8%)	-0.1% (-0.5–0.2%)	-0.708
Thromboembolic events	176(0.6%)	116(0.5%)	82 (0.5%)	79 (0.5%)	-0.0% (−0.2–0.1%)	-0.237

Squared sum of Z differences: 13.5. Number of co-variables: 30. Caliper width: 0.02.

*CI –* confidence interval, *LoC* – level of care, *PHF –* proximal humeral fracture, *SD –* standard deviation, *n.a*. – not applicable

The balance of the matched cohort is reported in Table 1 using SSQZ, Z differences and relative risk differences for all included co-variables after matching.

### Statistical analysis

For OS and LoC worsening including death, event rates were calculated as 1 minus the survival function determined using Kaplan-Meier estimates. In addition, hazard ratios (HRs) with unadjusted 95% confidence intervals (95% CI) were determined using Cox proportional hazard models. For all other primary endpoints, death was considered as a competing risk event, and event rates were given as cumulative incidence functions determined using Aalen-Johansen estimates. Sub-distributional HRs with unadjusted 95% CIs were determined using the Fine and Gray model. As a sufficient balance between the treatment groups was achieved (see Table 1), treatment effects were estimated using univariate models. The dependency of matched pairs was accounted for using stratified analysis for all endpoints. P-values comparing surgical and non-operative treatment were jointly adjusted using the Bonferroni-Holm method to control the family-wise error rate with respect to the multiple comparison problem. Adjusted p-values were compared with the overall significance level of 5%. Statistical analyses were performed using SAS Enterprise Guide Version 8.3 Update 2 (SAS Institute Inc., Cary, NC, USA) and R Version 4.2.0 (22.04.2022, R Foundation for statistical computing, Vienna, Austria).

## Results

In total, 54,595 patients (median age 79 years, 84% female) were included in the study. Surgical treatment was performed in 44.3% of all patients, with RTSA being the most frequently used treatment strategy (sLPF: 5.9%, LPF 30.9%, RTSA 32.2%, others 18.7%). Surgically treated patients less frequently had a pre-existing LoC (non-op without LoC 64.7%, op without LoC 74.4%) and were less often cared for in a nursing home (non-op 9.5% vs. op 4.9%). After 1:1 propensity score matching, *N* = 31,314 patients were included for further analyses (see Table 1).

### Deterioration of the level of care during follow-up

**Fig. 2:**
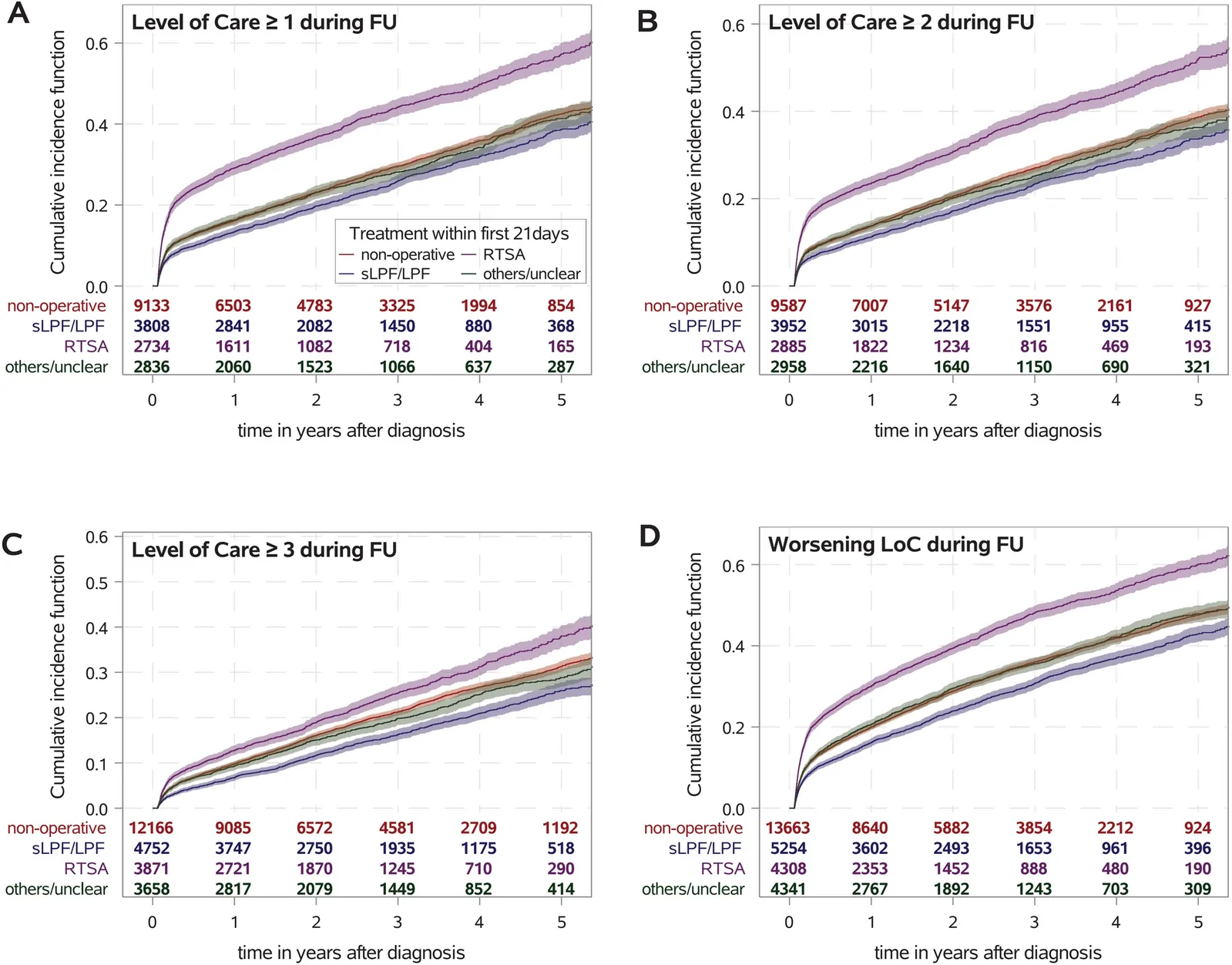
Cumulative incidence functions/event rates (95% confidence interval) for care-related outcomes determined by Aalen-Johansen estimates, with death being considered a competing risk event. Patients were classified by treatment group (locked plate fixation for simple and multi-fragmented fractures – sLPF/LPF; reverse total shoulder arthroplasty – RTSA; other open reduction and internal fixation procedures or unclear treatment – others/unclear) within 21 days after proximal humeral fracture. For stratified data, observation started 21 days after fracture. **A:** Level of Care (LoC) ≥ I during follow-up (FU), **B:** LoC ≥ II during FU. **C:** LoC ≥ III during FU. **D:** Worsening of LoC during FU. Detailed data visualized in Fig. 2, Fig. S2 and Fig. S3 can also be found in a tabular format in Table S2

**Fig. 3:**
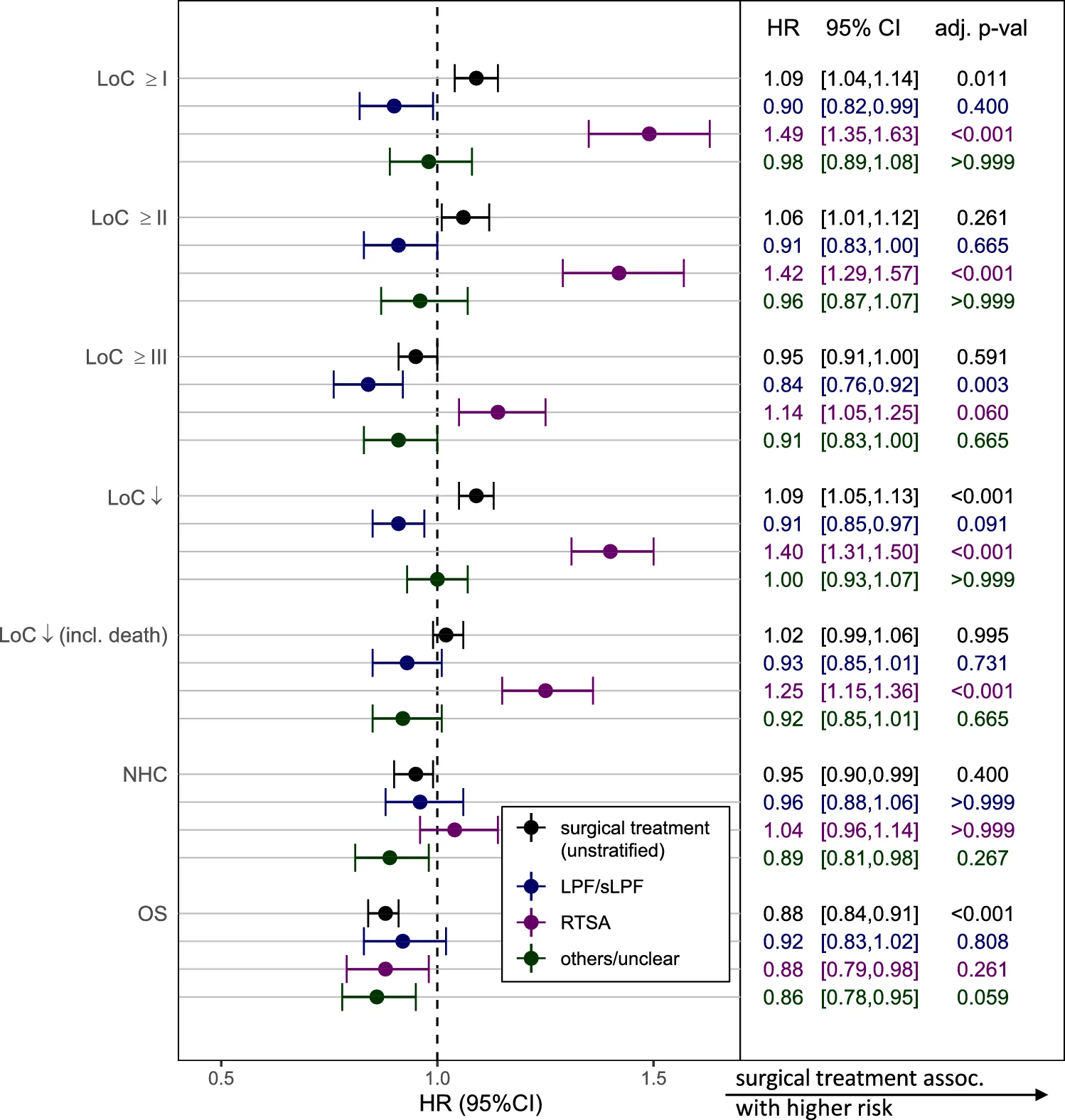
Multivariable analyses for primary and secondary endpoints. Overall survival (OS) and deterioration of the level of care (LoC↑) or death were modeled using a proportional hazards Cox model. For all other endpoints (LoC ≥ I; LoC ≥ II; LoC ≥ III; LoC↑; nursing home care – NHC), death was considered a competing risk event and sub-distributional hazard ratios (HRs) were determined using multivariable Fine and Gray models. In all models, clustering due to matching partners was considered. The treatment effect was evaluated by two models: First, including a binary variable for surgical treatment yes/no (marked as “unstratified”), and second, including a categorical variable with different treatment options (locked plate fixation for simple and multi-fragmented fractures – sLPF/LPF; reverse total shoulder arthroplasty – RTSA; other open reduction and internal fixation procedures or unclear treatment – others/unclear). All p-values for the treatment effect were jointly adjusted using the Bonferroni-Holm method

One year after fracture, 21.3% (20.8–21.8%) of the patients had a higher LoC than before PHF. In total, without stratification by different treatment strategies, surgical treatment was associated with a significantly higher risk of LoC deterioration (HR 1.09, 95% CI 1.05–1.13; *p < *0.001). However, when death was included, no risk differences were observed between surgical and non-operative treatment (*p = *0.995). Nevertheless, there were distinct differences between surgical treatment options (see Fig. 2, Figure S2, Figure S3): patients treated with RTSA showed a significantly higher risk of LoC deterioration compared to the non-operative treatment cohort (HR 1.40; 95% CI 1.31–1.50; *p < *0.001), while those treated with sLPF/LPF showed no significant difference compared to non-operatively treated patients (*p = *0.091; see Fig. 3). In a less pronounced manner, similar associations were observed for the composite endpoint of “deterioration of LoC or death” (see Fig. 3).

For patients treated with RTSA, a significantly increased risk was observed only for lower LoC, specifically, transitions from no LoC to LoC I (HR 1.49; 95% CI 1.35–1.63; *p < *0.001) and to LoC II (HR 1.42; 95%CI 1.29–1.57; *p < *0.001; see Fig. 3). Beyond this, no significant differences were seen in reaching severe impairment (i.e., LoC ≥ III) compared to non-operative treatment (*p = *0.060; see Fig. 3).

Three years after fracture, 20.8% (20.3–21.4%) of patients with low LoC (< III) progressed to LoC ≥ III, indicating severe impairment. Patients treated with sLPF/LPF showed a lower risk of developing severe impairment (i.e., LoC ≥ III) during follow-up compared to non-operatively treated patients (HR 0.84; 95%CI 0.76–0.92; *p = *0.003).

### Need for nursing home care

A total of 7.4% of patients (before matching) had already been cared for in a nursing home before the PHF. These patients were less often treated surgically (NHC patients: 29.0% vs. patients living at home: 45.6%). During follow-up, the proportion of patients (matched cohort) cared for in a nursing home increased substantially: 11.1% (10.7–11.4%) of those who had previously lived at home were cared for in a nursing home one year after PHF (see Table S2, Figure S2). However, no significant differences in the need for NHC between surgically and non-operatively treated patients were observed (*p = *0.4, see Fig. 3).

### Overall survival

One year after PHF, 10.8% (10.5–11.2%) of patients were deceased (see Table S2, Figure S3). Surgical treatment was associated with significantly lower overall mortality during follow-up (HR 0.88, 95% CI 0.84–0.91; *p < *0.001; see Figs. 3, S3).

## Discussion

### Main findings and clinical relevance

The present study provides a comprehensive analysis of treatment strategies and their impact on care dependency and overall survival in patients with PHF. From a geriatric perspective, our findings highlight PHF as a sentinel event that may initiate or accelerate trajectories of functional decline and care dependency. In our cohort, surgical treatment was performed in almost half of the cases, with RTSA being the most frequently chosen approach. Surgical treatment was associated with a higher risk of LoC deterioration compared to non-operative treatment, but with a lower overall mortality risk during follow-up. In particular, patients undergoing RTSA showed an increased risk of LoC deterioration. However, no differences in severe impairment (LoC ≥ III) or need for nursing home care were observed between surgical and non-operative treatment, although treatment-specific differences became apparent in subgroup analyses. Overall, about one-fifth of patients experienced a worsening of LoC one year after PHF. Furthermore, the proportion of patients requiring assisted living or nursing home care rose substantially during the first year, regardless of treatment method, underscoring the vulnerability of this population after a fracture event.

### Survival and long-term care dependency

Our findings are consistent with a study by Hammes et al*.,* which analyzed one-year outcomes following PHF using health insurance claims data from the German federal state of North Rhine-Westphalia [21]. The authors reported a 57% increase in the number of patients requiring professional care for the first time, and found that 19.5% of patients transitioned to nursing home care within one year post-fracture. They further reported a one-year mortality of 13% and identified a negative association between non-operative treatment and overall survival, which aligns with our results (1-year mortality: 10.8%) [21]. A report from Finland also showed that PHF increases the risk of mortality, with a standardized mortality ratio of 2.2 (95% CI: 1.8–2.6) [25]. In a Swedish study of 147,692 patients with PHF, the 1-year mortality rate after PHF was 8.5% [26]. A recent health claims study from Japan reported an overall 1-year mortality of 6.8% with a lower mortality rate in the surgical group than in the non-surgical group among patients 75–84 years old who required long-term care [27]. The same study also reported a worsening of long-term care in 20% of patients aged 65 years or older 1 year after PHF, which is consistent with our findings (LoC deterioration: 21.3%). Although propensity score matching resulted in well-balanced treatment groups, certain influencing factors, such as the surgeon’s experience and subjective assessment of the patient’s general health condition, cannot be fully addressed and may have contributed to the observed survival advantage in surgically treated patients. This reduced mortality is unlikely to be solely attributable to the surgical intervention itself. It may also reflect a selection bias, as patients chosen for surgery were potentially in better overall health or exhibited a lower degree of frailty, both of which are independently associated with a more favorable prognosis. However, surgical stabilization may also facilitate earlier mobilization, improved pain control, and restoration of upper limb function, potentially reducing complications associated with prolonged immobilization in older adults. Additionally, the observed LoC deterioration in 21.3% of the patients following PHF does not necessarily indicate a true decline in function. Rather, some patients may already have required a higher LoC before the fracture, with the fracture event itself triggering formal reassessment and subsequent application for additional care services. This interpretation is supported by the absence of significant differences in nursing home care requirements between surgical and non-operative treatment in the matched cohort. This further complicates the interpretation of the direct impact of PHF on long-term care dependency. Nonetheless, previous studies have shown that 17% [28] and 19.5% [21] of patients with PHF, respectively, had to give up living independently, highlighting the substantial impact of PHF on autonomy in older adults. One of these studies also reported a high incidence of “fear of falling” and impaired walking ability, leading to new falls in 28% of patients [28].

### Outcomes after RTSA and LPF

The increased risk of LoC deterioration following RTSA may indicate that, although this procedure offers mechanical stability and pain relief, it could also contribute to functional decline, particularly in vulnerable older patients. Functional recovery in this population is strongly influenced by baseline mobility, frailty, and the ability to resume activities of daily living, which may not be fully reflected by procedure-specific outcomes. Although the observed difference in severe impairment between RTSA and non-operative treatment did not reach statistical significance after adjustment for multiple comparisons (adjusted *p = *0.06), the unadjusted p-value was below the conventional threshold of 0.05, and the 95% CI was 1.14 [1.05, 1.25]. This may indicate a clinically relevant trend that may have failed to reach significance primarily due to limited statistical power. In contrast to our findings, recent studies have reported better clinical and functional outcomes after RTSA compared to LPF [6, 29], although high-level evidence remains limited [4].

Interestingly, patients treated with LPF did not exhibit an increased risk of LoC deterioration. This is in line with a previously published study by Ott et al*.,* which showed that significantly more patients treated with ORIF/LPF compared to RTSA were able to return to independent living and were discharged to their own homes [20]. In addition, surgery duration, length of hospital stay, and injury-related complication rates have been reported to be lower after LPF compared to RTSA [3, 20, 22, 30]. In a previous nationwide health claims analysis of more than 55,000 older patients with PHF, RTSA was associated with a substantially longer length of hospital stay compared to LPF (20.0 vs. 14.6 days), accompanied by significantly higher treatment costs and an increased impact of comorbidities and complications on hospitalization duration [30]. Longer hospital stays after RTSA may be clinically relevant in frail geriatric patients, as prolonged inpatient treatment is frequently associated with reduced mobility, deconditioning, and loss of independence. However, LPF also carries a higher risk of secondary revision surgery, which may increase complication rates [31].

### Clinical decision-making and rehabilitation strategies

As this nationwide health claims analysis does not contain detailed information on individual clinical decision-making processes or rehabilitation pathways, only general treatment and rehabilitation strategies routinely applied at our institution can be described. In routine clinical practice in Germany, treatment decisions are primarily based on an individualized assessment of the patient, including general condition, frailty, comorbidities, functional status, fracture morphology, and the surgeon’s clinical judgment and experience. To date, no evidence-based criteria directly link the pre-fracture LoC or burden of comorbidity to the choice of surgical procedure. Nevertheless, clinical experience plays an important role in decision-making, and patients with severe frailty or a high pre-existing LoC are often more likely to receive conservative treatment whenever feasible. Consequently, the decision for surgical versus conservative treatment is generally made on a case-by-case basis rather than according to standardized protocols. RTSA is typically preferred over LPF in older patients with poor bone quality and complex fracture patterns, particularly when stable fixation with osteosynthesis appears unlikely. In addition, RTSA is commonly selected in cases of irreparable humeral head damage, pre-existing rotator cuff dysfunction, or as a salvage procedure after failed conservative or operative treatment.

Standardized physiotherapy and rehabilitation protocols are routinely applied after both conservative and surgical treatment with LPF or RTSA, with rehabilitation strategies adapted to treatment modality and fracture stability. Conservative treatment generally includes temporary immobilization combined with early physiotherapy and progressive mobilization exercises. In contrast, postoperative rehabilitation after surgical treatment aims at earlier functional mobilization due to mechanical fracture stabilization, with shorter and less restrictive immobilization and early passive and assisted mobilization under physiotherapeutic supervision. After LPF, rehabilitation focuses on gradual mobilization while protecting the osteosynthesis, whereas after RTSA, prevention of prosthetic instability and dislocation represents an additional focus. During the inpatient stay, physiotherapy and mobilization are initiated immediately, followed by structured outpatient physiotherapy after discharge. Particularly in older and frail patients, transfer to a geriatric rehabilitation facility is pursued whenever clinically feasible to support functional recovery and preserve independence.

Taken together, these findings emphasize that treatment decisions after PHF have implications extending beyond fracture healing and shoulder function, directly affecting long-term autonomy and care dependency. Our results further highlight the complex relationship between treatment decisions and patient outcomes after PHF, as well as the potential implications for the German LTCI system. Currently, no clear consensus exists regarding the optimal treatment modality for older patients with PHF, although treatment algorithms based on clinical experience and factors, such as patient age, fracture complexity, and accompanying cuff arthropathies, are increasingly emerging [32]. An analysis of 7,012 case scenarios evaluated by 340 surgeons demonstrated substantial variation in treatment preferences across different age groups [32]. LPF emerged as the preferred modality for individuals younger than 50 years with complex fracture patterns, whereas both LPF and RTSA were commonly used in patients aged 50–70 years. In patients older than 70 years, surgeons predominantly favored RTSA, which corresponds with our findings that RTSA (32.2%) was the most frequently applied surgical treatment modality in this cohort of patients aged 65 years and older. From a geriatric perspective, this age-related shift in treatment preference raises the question of how adequately current decision-making captures functional reserve and vulnerability in older adults. Considering these observations, individualized treatment strategies remain essential to avoid poor functional outcomes in older patients with PHF. Future studies, ideally large prospective multicenter trials or registry-based analyses, are needed to confirm these findings and to further clarify the functional and care-related outcomes of different surgical strategies for PHF.

### Strengths and limitations

A key limitation of this study is the potential for selection bias in treatment decisions. While propensity score matching was applied to balance observed confounding factors, unobserved confounders, such as surgeon’s clinical experience and treatment preferences, may not be fully addressed. Furthermore, similar limitations are also present in randomized controlled trials, where strict inclusion and exclusion criteria often lead to an underrepresentation of frail patients or systematic exclusion of individuals at high risk of mortality. Additionally, the data were originally collected for economic purposes, introducing the possibility of upcoding, which could affect calculations and reported outcomes. Nevertheless, both treatment groups are likely affected equally. Furthermore, discrepancies between prescribed and actually taken medications, as well as the lack of detailed fracture classifications or documented treatment rationale, limit the interpretation of the findings. Despite these limitations, real-world data remain invaluable for assessing treatment strategies in routine clinical practice, especially in heterogeneous older populations that are often underrepresented in controlled trials.

## Conclusions

Our nationwide analysis demonstrates that treatment strategies for PHF in older patients are closely linked to care dependency and survival. From a geriatric perspective, these findings underline the relevance of PHF as an event with potential long-term consequences for patient independence and need for support. While RTSA may be associated with an increased risk of LoC deterioration, its impact on severe long-term impairment appears limited. Surgical treatment overall was linked to lower mortality and LPF did not show an increased risk of care dependency. Importantly, lower mortality does not necessarily translate into preserved independence, highlighting the need to consider functional and care-related outcomes alongside survival. These findings support the need for individualized treatment decisions that balance functional outcomes, pre-existing care needs, frailty, and mortality risk, in line with the core principles of geriatric medicine.

## Supplementary Information

Below is the link to the electronic supplementary material.

Supplementary file1 (DOCX 583 KB)

## Data Availability

The authors state that the data utilized in this study cannot be made available in the manuscript, the additional files, or in a public repository due to German data protection laws (‘Bundesdatenschutzgesetz’, BDSG). They are stored on a server of the BARMER Institute for Health System Research, to facilitate replication of the results. In general, access to data from statutory health insurance funds for research purposes is possible only under the conditions defined in German Social Law (SGB V §287).
