# Supplementary material for: How does the level of care change after proximal humeral fracture in older patients? A propensity score matching analysis
Source: Eur Geriatr Med. 2026 Jun 26;17(4):2163–73. doi: 10.1007/s41999-026-01531-w (PMC13562273; doi:10.1007/s41999-026-01531-w)
Supplement: Supplementary file 1 [file 41999_2026_1531_MOESM1_ESM.docx]

**Supplemental Material**

**Data availability statement:**

The authors confirm that the data utilized in this study cannot be made available in the manuscript, the supplemental files, or in a public repository due to German data protection laws (‘Bundesdatenschutzgesetz’, BDSG). They are stored on a server of the BARMER Institute for Health System Research to facilitate replication of the results. In general, access to data from statutory health insurance funds for research purposes is possible only under the conditions defined in German Social Law (SGB V, § 287).

**Additional Information 1**

*The German LTCI system*

In Germany, the statutory long-term care insurance (LTCI) system defines five levels of care (“Pflegegrade”), ranging from Level I (minor impairment) to Level V (most severe impairment). These levels are granted following a standardized medical assessment and reflect a patient’s ability to perform activities of daily living and the extent of support required:

- LoC I: Minor limitations in independence with occasional assistance needed.
- LoC II: Significant impairments requiring daily support.
- LoC III: Severe impairments requiring extensive assistance with most activities.
- LoC IV: Very severe impairments with patients often depending on comprehensive daily care, often living in nursing home care.
- LoC V: Most severe impairments with patients often bedbound or wheelchair-bound, requiring full-time nursing care, often living in nursing home care.

Assignment to a LoC determines the amount of financial support and care services covered by LTCI. For this study, information on pre-existing LoC was extracted from the claims data, which systematically records the LTCI level granted to each insured person.

**Supplementary Figure S1:** Consort flow chart. PHF – proximal humeral fracture. PS – propensity score.

**Supplementary Figure S2:** Cumulative incidence functions/event rates (95% confidence interval) for the need for nursing home care during follow-up, determined by Aalen-Johansen estimates, with death being considered a competing risk event. Patients were classified by treatment group within 21 days after proximal humeral fracture, i.e. for stratified data, observation started 21 days after fracture.

**Supplementary Figure S3:** Overall survival after proximal humeral fracture. The survival function was determined using Kaplan-Meier estimates and is presented with 95% confidence intervals. Patients were classified by treatment group within 21 days after proximal humeral fracture, i.e. for stratified data, observation started 21 days after fracture.

**Supplementary Table S1:** Definition of all variables, including diagnosis, procedure, and pharmaceutical codes, as well as endpoints. LPF – locked plate fixation, PHF – proximal humeral fracture, RTSA – reverse total shoulder arthroplasty.

^1^Data collection is based on outpatient and inpatient information within the period of two years before the index event (i.e., first coded diagnosis of PHF). Exception: Rotator cuff rupture – only if coded at the index event.

^2^Only based on outpatient data after PHF.

| Variable | Code | Codes |
| --- | --- | --- |
| Proximal humeral fracture | ICD | **S42.2** |
| Simple fracture LPF | OPS | **5-793.k1, 5-793.31** |
| Multi-fragmented LPF | OPS | **5-794.21, 5-794.k1** |
| RTSA | OPS | **5-824.21** |
| Other fracture fixation | OPS | **5-790.01**, 5-790.11, 5-790.21, 5-790.31, 5-790.41,  5-790.51, 5-790.61, 5-790.71, 5-790.81, 5-790.91,  5-790.d1, 5-790.m1, 5-790.n1, 5-790.p1, 5-790.x1,  **5-793.11**, 5-793.21, 5-793.41, 5-793.51, 5-793.61,  5-793.71, 5-793.81, 5-793.91, 5-793.a1, 5-793.b1,  5-793.c1, 5-793.g1, 5-793.m1, 5-793.n1, 5-793.x1,  **5-794.01**, 5-794.11, 5-794.31, 5-794.41, 5-794.51,  5-794.61, 5-794.71, 5-794.81, 5-794.a1, 5-794.b1,  5-794.c1, 5-794.g1, 5-794.m1, 5-794.n1, 5-794.x1,  **5-824.00**, 5-824.01, 5-824.0x, 5-824.20 |
| Comorbidities at index^1^ | | |
| Alcohol abuses | ICD | **E**24.4, **F**10, **G**31.2, G62.1, G72.1, **I**42.6, **K**29.2, K70, K85.2, K86.0, **T**51.0, T51.9 |
| Atherosclerosis | ICD | I70 |
| Atrial fibrillation and flutter | ICD | I48 |
| Bone tumor/metastasis | ICD | C40.0, C79.5, C79.86, C79.9 |
| Cancer | ICD | C |
| Chronic kidney disease | ICD | N18, N19 |
| Chronic polyarthritis | ICD | M05, M06 |
| Congestive heart failure | ICD | I50 |
| Coronary heart disease | ICD | I25 |
| Dementia | ICD | **F**00, F01, F02, F051, **G**30, G31.1 |
| Diabetes mellitus | ICD | E10 – E14 |
| Hypertension | ICD | I10 – I15 |
| Infection | ICD | **M**86.01, M86.11, M86.21, M86.31, M86.41, M86.51, M86.61, M86.81, M86.91, **T**84.5, T84.6, T84.7 |
| Nicotine abuses | ICD | F17 |
| Obesity | ICD | E66 |
| Osteoporosis | ICD | M80 – M85 |
| Parkinson | ICD | G20 |
| Polytrauma | ICD, OPS, DRG | ICD: T07; OPS: 5-982, or coded DRG started with “W” |
| Rotator cuff rupture | ICD | M75.1, S46.0 |
| Omarthrosis | ICD | M25.51 |
| Previous stroke and other cerebrovascular disease | ICD | I60 – I69 |
| Pharmaceutical therapy | | |
| Any anticoagulant | ATC | B01AA, B01AB, B01AC, B01AE, B01AF, B01AX |
| Vitamin D or calcium | ATC | A11CC |
| Bisphosphonates | ATC | M05BA, M05BB |
| Any osteoporosis pharmacotherapy | ATC | Vitamin D/Calcium or Bisphosphonates |
| Osteoporosis associated fractures | | |
| Distal radius | ICD | S52.2, S52.6 |
| Proximal femur | ICD | S72.0, S72.1 |
| vertebral fracture | ICD | S12.0, S12.1, S12.2, S12.7, S12.9, S22.0, S32.0 |
| pelvic ring fractures | ICD | S32.1, S32.2 S32.3, S32.5, S32.8.1 |
| Osteoporosis with pathologic fracture | ICD | M80 |
| General complications^2^ | | |
| Acute liver failure | ICD | K72.0, K72.7, K72.9 |
| Acute myocardial infarction | ICD | I21, I22 |
| Acute renal failure | ICD | N17 |
| Acute respiratory distress syndrome | ICD | J80 |
| Cardiac arrest | ICD | I46 |
| Deep vein thrombosis | ICD | I80.1, I80.2, I82.2, I82.3 |
| Pulmonary embolism | ICD | I26 |
| Sepsis | ICD | A41, A40, B37.7, R65.0, R65.1, R65.9, R57.2 |
| Stroke | ICD | I60 – I64 |
| Injury-related or surgical complications^2^ | | |
| Failure of non-surgical treatment | OPS | **5-78**5.01, 5-785.11, 5-785.21, 5-785.51, 5-785.61,  5-785.71, **5-789**.31, **5-78a**.01, 5-78a.11, 5-78a.21,  5-78a.31, 5-78a.41, 5-78a.51, 5-78a.61, 5-78a.71,  5-78a.90, 5-78a.c1, 5-78a.g1, 5-78a.k1, **5-790.01,**  5-790.11, 5-790.21, 5-790.31, 5-790.41, 5-790.51,  5-790.61, 5-790.71, 5-790.81, 5-790.91, 5-790.d1,  5-790.m1, 5-790.n1, 5-790.p1, 5-790.x1, **5-793**.01,  5-793.11, 5-793.21, 5-793.31, 5-793.41, 5-793.51,  5-793.81, 5-793.91, 5-793.c1, 5-793.g1, 5-793.a1,  5-793.b1, 5-793.h1, 5-793.k1, 5-793.m1, 5-793.n1,  5-793.x1, **5-794**.01, 5-794.11, 5-794.21, 5-794.31,  5-794.41, 5-794.51, 5-794.61, 5-794.71, 5-794.81,  5-794.a1, 5-794.b1, 5-794.c1, 5-794.g1, 5-794.k1,  5-794.m1, 5-794.n1, 5-794.x1, **5-824**.00**,** 5-824.01,  5-824.21, 5-824.0x |
| Upper limb amputation, ipsilateral (shoulder or upper arm) | OPS | **5-862**.1, 5-862.2 |
| Delayed union (LPF and non-surgical treatment; if coded within six months) | OPS | **5-781**.a1, **5-782**.11, 5-782.21, 5-782.31, 5-782.41,  5-782.51, 5-782.61, 5-782.a1, 5-782.b1, **5-784**.01,  5-784.11, 5-784.21, 5-784.31, 5-784.41, 5-784.71,  5-784.81, 5-784.b1 |
| Infection | OPS | **5-780**.41, 5-780.51, 5-780.61, 5-780.71, 5-780.81,  5-780.91, **5-800**.20, 5-800.30, 5-800.a0, 5-800.b0,  **5-810**.10, 5-810.70, 5-810.80, **8-989**, 8-989.0, 8-989.1, 8-989.2, 8-989.3, 8-989.4, 8-989.5, 8-989.6 |
| Joint damage / cartilage damage (LPF and non-surgical treatment) | OPS | 5-780.31, 5-784.51, 5-784.61, 5-800.80, 5-810.40,  5-812.00, 5-812.30, 5-812.90, 5-812.a0, 5-812.e0,  5-812.f0, 5-812.g0, 5-812.h0, 5-812.k0, 5-812.m0 |
| Luxation | OPS | **8-201.0** |
| Malunion (LPF and non-surgical treatment) | OPS | **5-781**.01, 5-781.11, 5-781.21, 5-781.31, 5-781.41,  5-781.51, 5-781.61, 5-781.81, 5-781.91 |
| Nerve injury | OPS | **5-040**.1, 5-040.2, 5-040.3, **5-041**.1, 5-041.2, 5-041.3,  **5-044**.1, 5-044.2, 5-044.3, **5-045**.1, 5-045.2, 5-045.3,  **5-046**.1, 5-046.2, 5-046.3, **5-047**.1, 5-047.2, 5-047.3,  **5-048**.1, 5-048.2, 5-048.3, **5-049**.1, 5-049.2, 5-049.3,  **5-04b**.1, 5-04b.2, 5-04b.3, **5-050**.1, 5-050.2, 5-050.3,  **5-051**.1, 5-051.2, 5-051.3, **5-052**.1, 5-052.2, 5-052.3,  **5-053**.1, 5-053.2, 5-053.3, **5-054**.1, 5-054.2, 5-054.3,  **5-055**.1, 5-055.2, 5-055.3, **5-056**.1, 5-056.2, 5-056.3,  **5-057**.1, 5-057.2, 5-057.3 |
| Non-union / Pseudoarthrosis (LPF and non-surgical treatment; if coded after six months) | OPS | **5-781**.a1, **5-782**.11, 5-782.21, 5-782.31, 5-782.41,  5-782.51, 5-782.61, 5-782.a1, 5-782.b1, **5-784**.01,  5-784.11, 5-784.21, 5-784.31, 5-784.41, 5-784.71,  5-784.81, 5-784.b1 |
| Osteonecrosis (LPF and non-surgical treatment) | ICD | **M87.21, M87.22, M87.32, M87.82, M87.92** |
| Postoperative stiffness, Adhesive capsulitis, Frozen shoulder | OPS | **5-800**.60, 5-800.c0, **5-810**.20, 5-810.90 |
| Vascular injury | OPS | **5-388**.11, 5-388.12, **5-395**.11, 5-395.12, **5-397**.11,  5-397.12 |
| Secondary surgery, open (LPF, non-surgical treatment) | OPS | **5-780**.01, 5-780.11, 5-780.21, 5-780.31, 5-780.61,  5-780.x1, **5-782**.11, 5-782.21, 5-782.31, 5-782.41,  5-782.51, 5-782.62, 5-782.72, 5-782.82, 5-782.92,  5-782.a1, **5-784**.01, 5-784.11, 5-784.21, 5-784.31,  5-784.41, 5-784.51, 5-784.61, 5-784.71, 5-784.81,  5-784.a1, 5-784.b1, **5-785**.01, 5-785.11, 5-785.21,  5-785.31, 5-785.41, 5-785.51, 5-785.61, 5-785.71,  **5-789**.b1, 5-789.c1, **5-794**.01, 5-794.11, 5-794.21,  5-794.31, 5-794.41, 5-794.71, 5-794.81, 5-794.k1,  **5-800**.10, 5-800.30, 5-800.40, 5-800.50, 5-800.70,  5-800.80, 5-800.90, 5-800.x0, **5-801**.00, 5-801.30,  5-801.40, 5-801.b0, 5-801.c0, 5-801.g0, 5-801.h0,  5-801.k0, 5-801.m0, 5-801.n0, 5-801.p0, **5-805**.0,  5-805.1, 5-805.2, 5-805.3, 5-805.4, 5-805.5, 5-805.6,  5-805.7, 5-805.8, 5-805.9, 5-805.a, **5-850**.01, 5-850.11, 5-850.21, 5-850.31, 5-850.41, 5-850.51, 5-850.61,  5-850.71, 5-850.81, 5-850.91, 5-850.a1, 5-850.b1,  5-850.c1, 5-850.d1, 5-850.e1, 5-850.f1, 5-850.g1,  5-850.h1, 5-850.j1, 5-850.x1, **5-851**.11, 5-851.21,  **5-852**.01, 5-852.11, **5-853**.01, 5-853.11, **5-855**.01,  5-855.11, 5-855.21, 5-855.51, 5-855.61, 5-855.71,  5-855.81, 5-855.91, 5-855.a1, **5-859**.01, 5-859.11,  **5-862**.1, 5-862.2, **5-892**.06, 5-892.07, 5-892.16,  5-892.17, **5-896**.06, 5-896.16, 5-896.26 |
| Secondary surgery, open (RTSA) | OPS | **5-780**.01, 5-780.11, 5-780.21, 5-780.31, 5-780.61,  5-780.x1, **5-782**.11, 5-782.21, 5-782.31, 5-782.41,  5-782.51, 5-782.62, 5-782.72, 5-782.82, 5-782.92,  5-782.a1, **5-785**.01, 5-785.11, 5-785.21, 5-785.31,  5-785.41, 5-785.51, 5-785.61, 5-785.71, **5-789**.b1,  **5-791**.02, 5-791.12, 5-791.22, **5-792**.02, 5-792.12,  5-792.22, 5-792.k2, **5-800**.10, 5-800.30, 5-800.40,  5-800.50, 5-800.70, 5-800.80, 5-800.90, 5-800.x0,  **5-850**.01, 5-850.11, 5-850.21, 5-850.31, 5-850.41,  5-850.51, 5-850.61, 5-850.71, 5-850.81, 5-850.91,  5-850.a1, 5-850.b1, 5-850.c1, 5-850.d1, 5-850.e1,  5-850.f1, 5-850.g1, 5-850.h1, 5-850.j1, 5-850.x1,  **5-859**.01, 5-859.11, **5-862**.1, 5-862.2, **5-892**.06,  5-892.07, 5-892.16, 5-892.17, **5-896**.06, 5-896.16,  5-896.26 |
| Secondary arthroscopy (LPF, non-surgical treatment) | OPS | **5-782**.b1, **5-784**.c1, 5-784.d1, 5-784.e1, 5-784.f1,  **5-810**.00, 5-810.10, 5-810.20, 5-810.40, 5-810.50,  5-810.70, 5-810.80, 5-810.90, **5-811**.20, 5-811.30,  5-811.40, **5-812**.00, 5-812.30, 5-812.40, 5-812.90,  5-812.a0, 5-812.e0, 5-812.f0, 5-812.g0, 5-812.h0,  5-812.k0, 5-812.m0, **5-814**.0, 5-814.1, 5-814.2, 5-814.3, 5-814.4, 5-814.5, 5-814.6, 5-814.7, 5-814.8, 5-814.9,  5-814.b, 5-814.c, 5-814.d, 5-814.e, **5-819**.00, 5-819.10, 5-819.20 |
| Secondary arthroscopy (RTSA) | OPS | **5-782**.b1, **5-810**.00, 5-810.10, 5-810.20, 5-810.40,  5-810.50, 5-810.70, 5-810.80, 5-810.90, **5-811**.20,  5-811.30, 5-811.40, **5-814**.b, 5-814.c, 5-814.d, 5-814.e, **5-819**.00, 5-819.10, 5-819.20 |
| Revision (LPF) | OPS | **5-78**5.01, 5-785.11, 5-785.21, 5-785.51, 5-785.61,  5-785.71 **5-789**.31, 5-78a.01, 5-78a.11, 5-78a.21,  5-78a.31, 5-78a.41, 5-78a.51, 5-78a.61, 5-78a.71,  5-78a.90, 5-78a.c1, 5-78a.g1, 5-78a.k1, **5-793**.01,  5-793.11, 5-793.21, 5-793.31, 5-793.41, 5-793.51,  5-793.81, 5-793.91, 5-793.c1, 5-793.g1, 5-793.a1,  5-793.b1, 5-793.h1, 5-793.k1, 5-794.01, **5-794**.11,  5-794.21, 5-794.31, 5-794.41, 5-794.71, 5-794.81,  5-794.k1, **5-824**.01, 5-824.21 |
| Revision (RTSA) | OPS | **5-785**.01, 5-785.11, **5-787**.01, 5-787.11, 5-787.21,  5-787.31, 5-787.k1, **5-789**.31, **5-78a**.01, 5-78a.11,  5-78a.21, 5-78a.k1, **5-810**.40, **5-824**.21, **5-825**.00,  5-825.1 (only 2010-2012), 5-825.21, 5-825.8, 5-825.k, 5-825.k0, 5-825.k1, 5-825.kx |
| Resection arthroplasty (for spacer placement) | OPS | **5-829.3** |
| Secondary arthroplasty (LPF and non-surgical treatment) | OPS | **5-824**.0, 5-824.20, 5-824.21 |
| Arthrolysis (LPF and non-surgical treatment) | OPS | **5-800**.60, **5-810**.20, 5-810.90 |
| Decompression of subacromial space (LPF and non-surgical treatment) | OPS | **5-814.3** |
| Debridement (LPF and non-surgical treatment) | OPS | **5-819**.10 |
| Endpoints | | |
| Major adverse events |  | resuscitation, cardiac arrest, myocardial infarction, stroke, acute renal failure, acute liver failure, acute respiratory distress syndrome, sepsis or death from any cause |
| Thromboembolic event |  | Deep vein thrombosis, pulmonary embolism, ischemic stroke or death from any cause |
| Surgical complications |  | Adhesive capsulitis, arthrolysis, conversion debridement, decompression of subacromial space, frozen shoulder, infection, infection with antibiotic-resistant germs, joint damage/cartilage damage, luxation, delayed union, non-union/ pseudoarthrosis, malunion, nerve injury, vascular injury, osteonecrosis, postoperative stiffness, secondary arthroplasty, secondary arthroscopy, secondary surgery (open) including revision surgery, upper limb amputation |
| Minor outpatient complications  (LPF) | ICD | **G**56.1, G56.2, G56.3, **I**80.80, I80.81, **M**00.01, M00.11, M00.21, M00.81, M00.91, M13.11, M13.81, M13.91, M19.11, M24.01, M24.11, M24.21, M24.41, M24.31, M24.51, M24.61, M25.11, M25.21, M25.31, M25.41, M25.51, M25.61, M25.71, M61.01, M62.21, M62.22, M62.41, M62.51, M62.61, M65.81, M65.91, M75.0, M75.1, M75.2, M75.4, M75.5, M84.21, M84.31, M86.01, M86.11, M86.21, M86.31, M86.41, M86.51, M86.61, M86.81, M86.91, M87.21, M87.22M87.31, M87.81, M87.91, M89.51, M96.6, **T**79.60, T84.10, T84.5, T84.6, T84.7 |
| Minor outpatient complications  (RTSA) | ICD | **G**56.1, G56.2, G56.3, **I**80.80, I80.81, **M**00.01, M00.11, M00.21, M00.81, M00.91, M24.21, M24.41, M24.31, M24.51, M24.61, M25.11, M25.21, M25.31, M25.41, M25.51, M25.61, M25.71, M61.01, M62.21, M62.41, M62.51, M62.61, M65.81, M65.91, M75.0, M75.1, M75.2, M75.4, M75.5, M84.31, M86.01, M86.11, M86.21, M86.31, M86.41, M86.51, M86.61, M86.81, M86.91, M96.6, **T**79.60, T81.4, T84.5, T84.6, T84.7, T84.00 |
| Minor outpatient complications  (non-surgical treatment) | ICD | **G**56.1, G56.2, G56.3, **I**80.80, I80.81, **M**00.01, M00.11, M00.21, M00.81, M00.91, M13.11, M13.81, M13.91, M19.11, M24.01, M24.11, M24.21, M24.41, M24.31, M24.51, M24.61, M25.11, M25.21, M25.31, M25.41, M25.51, M25.61, M25.71, M61.01, M62.21, M62.22, M62.41, M62.51, M62.61, M65.81, M65.91, M75.0, M75.1, M75.2, M75.4, M75.5, M84.21, M84.31, M86.01, M86.11, M86.21, M86.31, M86.41, M86.51, M86.61, M86.81, M86.91, M87.21, M87.22, M87.32, M87.82, M87.92, M89.51, M96.6, **T**79.60 |

**Supplementary Table S2:** Cumulative incidence functions/event rates (95% confidence interval) for care-related outcomes determined by Aalen-Johansen estimates, with death being considered a competing risk event. Patients were classified by treatment group within 21 days after proximal humeral fracture. These data were visualized in Figure 2, Figure S2 and Figure S3.

|  | Entire Cohort  (n= 31,314) | Non-operative treatment  (n= 15,657) | Surgical treatment | | | |
| --- | --- | --- | --- | --- | --- | --- |
|  |  |  | **any**  **(n= 15,657)** | **sLPF/LPF**  **(n= 5,820)** | **RTSA**  **(n= 4,980)** | **Others/unclear**  **(n= 4,857)** |
| PG ≥ I after PHF  1 year rate  2 years rate  3 years rate  4 years rate  5 years rate  total no. of events | 18.0%  (17.4 – 18.5%)  24.8%  (24.1 – 25.4%)  31.0%  (30.3 – 31.8%)  37.1%  (36.3 – 38.0%)  43.9%  (42.9 – 44.9%)  5,925 | 16.6%  (15.9 – 17.4%)  23.5%  (22.6 – 2447%)  29.9%  (28.9 – 31.0%)  36.2%  (35.0 – 37.4%)  42.8%  (41.4 – 44.2%)  2,831 | 19.3%  (18.5 – 20.1%)  26.1%  (25.1 – 27.0%)  32.1%  (31.1 – 33.2%)  38.0%  (36.8 – 39.2%)  45.0%  (43.5 – 46.4%)  3,094 | 13.3%  (12.2 – 14.4%)  19.4%  (18.1 – 20.8%)  25.9%  (24.3 – 27.5%)  31.7%  (29.9 – 33.6%)  37.7%  (35.5 – 40.0%)  1,028 | 29.8%  (28.0 – 31.5%)  37.3%  (35.3 – 39.2%)  44.6%  (42.5 – 46.7%)  50.2%  (47.8 – 52.5%)  57.7%  (54.8 – 60.4%)  1,196 | 16.7%  (15.4 – 18.1%)  23.6%  (21.9 – 25.2%)  28.5%  (26.7 – 30.3%)  34.5%  (32.4 – 36.6%)  41.6%  (39.1 – 44.2%)  870 |
| PG ≥ II after PHF  1 year rate  2 years rate  3 years rate  4 years rate  5 years rate  total no. of events | 15.1%  (14.6 – 15.6%)  21.7%  (21.1 – 22.3%)  28.0%  (27.3 – 28.7%)  33.5%  (32.7 – 34.3%)  39.5%  (38.5 – 40.4%)  5,508 | 14.1%  (13.4 – 14.9%)  20.9%  (20.0 – 21.7%)  27.3%  (26.3 – 28.3%)  32.9%  (31.8 – 34.0%)  39.0%  (37.6 – 40.3%)  2,661 | 16.0%  (15.3 – 16.8%)  22.4%  (21.6 – 23.3%)  28.7%  (27.7 – 29.7%)  34.1%  (32.9 – 35.2%)  40.0%  (38.6 – 41.3%)  2,847 | 11.5%  (10.5 – 12.5%)  17.5%  (16.2 – 18.8%)  23.7%  (22.2 – 25.2%)  28.4%  (26.7 – 30.2%)  33.9%  (31.8 – 36.0%)  945 | 24.2%  (22.7 – 25.8%)  31.3%  (29.5 – 33.1%)  39.1%  (37.1 – 41.2%)  44.8%  (42.5 – 47.0%)  52.5%  (49.7 – 55.3%)  1,100 | 14.0%  (12.8 – 15.3%)  20.4%  (18.9 – 22.0%)  25.5%  (23.8 – 27.2%)  31.6%  (29.6 – 33.6%)  36.5%  (34.2 – 38.9%)  802 |
| PG ≥ III after PHF  1 year rate  2 years rate  3 years rate  4 years rate  5 years rate  total no. of events | 9.8%  (9.4 – 10.1%)  15.6%  (15.1 – 16.1%)  20.8%  (20.3 – 21.4%)  26.2%  (25.5 – 26.9%)  31.0%  (30.1 – 31.8%)  5,163 | 9.9%  (9.4 – 10.4%)  16.1%  (15.4 – 16.8%)  21.3%  (20.5 – 22.2%)  26.9%  (25.9 – 27.8%)  34.1%  (30.2 – 32.6%)  2,641 | 9.6%  (9.1 – 10.1%)  15.1%  (14.4 – 15.8%)  20.3%  (19.5 – 21.1%)  25.5%  (24.5 – 26.5%)  30.6%  (29.4 – 31.8%)  2,522 | 6.9%  (6.2 – 7.7%)  11.8%  (10.8 – 12.8%)  16.4%  (15.3 – 17.7%)  21.1%  (19.7 – 22.6%)  26.1%  (24.3 – 27.9%)  806 | 13.1%  (12.0 – 14.2%)  19.2%  (17.9 – 20.5%)  25.7%  (24.1 – 27.3%)  31.5%  (29.6 – 33.4%)  38.3%  (35.9 – 40.6%)  963 | 9.5%  (8.5 – 10.5%)  15.2%  (14.0 – 16.4%)  19.9%  (18.5 – 21.4%)  25.2%  (23.5 – 27.0%)  28.9%  (26.9 – 31.0%)  753 |
| Worsening of PG after PHF  1 year rate  2 years rate  3 years rate  4 years rate  5 years rate  total no. of events | 21.3%  (20.8 – 21.8%)  29.9%  (29.4 – 30.5%)  37.0%  (36.4 – 37.7%)  43.0%  (42.3 – 43.7%)  48.8%  (47.9 – 49.6%)  10,040 | 20.3%  (19.6 – 21.0%)  29.1%  (28.3 – 29.9%)  36.3%  (35.4 – 37.2%)  42.1%  (41.1 – 43.1%)  47.7%  (46.6 – 48.9%)  4,865 | 22.2%  (21.5 – 22.9%)  30.8%  (30.0 – 31.6%)  37.8%  (36.8 – 38.7%)  43.9%  (42.9 – 44.9%)  49.8%  (48.6 – 51.0%)  5,175 | 16.4%  (15.4 – 17.4%)  24.2%  (23.0 – 25.4%)  30.9%  (29.5 – 32.3%)  37.2%  (35.6 – 38.8%)  43.1%  (41.1 – 44.9%)  1,627 | 30.6%  (29.2 – 32.0%)  39.9%  (38.3 – 41.4%)  48.4%  (46.7 – 50.1%)  53.9%  (52.0 – 55.8%)  60.3%  (58.1 – 62.5%)  1,964 | 21.0%  (19.8 – 22.2%)  29.8%  (28.4 – 31.3%)  35.8%  (34.3 – 37.4%)  42.4%  (40.6 – 44.2%)  48.1%  (46.0 – 50.1%)  1,584 |
| Worsening of PG (incl. death) after PHF  1 year rate  2 years rate  3 years rate  4 years rate  5 years rate  total no. of events | 27.3%  (26.7 – 27.8%)  38.3%  (37.7 – 39.0%)  47.1%  (46.4 – 47.7%)  54.4%  (53.7 – 55.2%)  61.1%  (60.3 – 62.0%)  12,699 | 26.8%  (26.1 – 27.6%)  38.1%  (37.2 – 38.9%)  46.8%  (45.8 – 47.7%)  54.1%  (53.0 – 55.1%)  60.5% (59.3 – 61.7%)  6,254 | 27.6%  (27.0 – 28.5%)  38.6%  (37.8 – 39.5%)  47.3%  (46.4 – 48.3%)  54.8%  (53.7 – 55.8%)  61.7%  (60.5 – 62.9%)  6,445 | 21.5%  (20.3 – 22.6%)  31.6%  (30.3 – 33.0%)  40.1%  (38.6 – 41.6%)  48.0%  (46.4 – 49.8%)  55.1%  (53.1 – 57.1%)  2,103 | 35.3%  (33.9 – 36.8%)  46.7%  (45.1 – 48.3%)  56.8%  (55.0 – 58.5%)  63.3%  (61.4 – 65.2%)  70.9%  (68.7 – 73.1%)  2,299 | 27.8%  (26.5 – 29.2%)  39.1%  (37.6 – 40.7%)  47.1%  (45.4 – 48.7%)  54.7%  (52.9 – 56.5%)  61.1%  (59.0 – 63.2%)  2,043 |
| Need of nursing home after PHF  1 year rate  2 years rate  3 years rate  4 years rate  5 years rate  total no. of events | 11.1%  (10.7 – 11.4%)  14.1%  (13.7 – 14.6%)  17.0%  (16.5 – 17.4%)  19.9%  (19.4 – 20.5%)  22.3%  (21.7 – 23.0%)  4,898 | 11.4%  (10.9 – 11.9%)  14.5%  (13.9 – 15.1%)  17.2%  (16.5 – 17.9%)  20.2%  (19.4 – 21.0%)  22.6%  (21.7 – 23.5%)  2,492 | 10.7%  (10.2 – 11.2%)  13.8%  (13.2 – 14.4%)  16.7%  (16.1 – 17.4%)  19.6%  (18.9 – 20.4%)  22.0%  (21.1 – 22.9%)  2,406 | 8.9%  (8.2 – 9.7%)  11.5%  (10.6 – 12.4%)  13.8%  (12.8 – 14.8%)  16.1%  (15.0 – 17.2%)  18.4%  (17.1 – 19.8%)  758 | 13.5%  (12.6 – 14.5%)  17.0%  (15.9 – 18.2%)  20.4%  (19.2 – 21.8%)  23.8%  (22.3 – 25.3%)  27.2%  (25.4 – 29.0%)  920 | 10.0%  (9.1 – 10.9%)  13.3%  (12.2 – 14.3%)  16.67%  (15.4 – 17.8%)  19.8%  (18.4 – 21.2%)  21.4%  (19.9 – 23.0%)  728 |
| Overall mortality  1 year rate  2 years rate  3 years rate  4 years rate  5 years rate  total no. of events | 10.8%  (10.5 – 11.2%)  17.8%  (17.4 – 18.3%)  24.7%  (24.2 – 25.3%)  31.7%  (31.0 – 32.3%)  37.9%  (37.1 – 38.7%)  7,811 | 11.5%  (11.0 – 12.0%)  18.8%  (18.2 – 19.5%)  25.7%  (24.9 – 26.5%)  32.7%  (31.8 – 33.6%)  39.2%  (38.0 – 40.3%)  4,067 | 10.1%  (9.6 – 10.6%)  16.8%  (16.2 – 17.5%)  23.8%  (23.0 – 24.6%)  30.6%  (29.7 – 31.5%)  36.6%  (35.4 – 37.6%)  3,744 | 8.4%  (7.7 – 9.2%)  14.7%  (13.8 – 15.7%)  20.9%  (19.7 – 22.1%)  27.2%  (25.7 – 28.6%)  33.0%  (31.3 – 34.8%)  1,263 | 10.2%  (9.4% – 11.1%)  17.1%  (15.9% – 18.2%)  24.9%  (23.4% – 26.3%)  31.9%  (30.2% – 33.7%)  39.8%  (37.5% – 42.0%)  1,183 | 11.9%  (11.0 – 12.9%)  19.0%  (17.9 – 20.2%)  26.2%  (24.8 – 27.7%)  33.5%  (31.8 – 35.2%)  38.2%  (36.1 – 40.0%)  1,298 |
